# Mixing mixed-mode designs in a national health interview survey: a pilot study to assess the impact on the self-administered questionnaire non-response

**DOI:** 10.1186/s12874-019-0860-3

**Published:** 2019-11-21

**Authors:** Elise Braekman, Sabine Drieskens, Rana Charafeddine, Stefaan Demarest, Finaba Berete, Lydia Gisle, Jean Tafforeau, Johan Van der Heyden, Guido Van Hal

**Affiliations:** 1 Scientific Direction Epidemiology and public health, Sciensano, Rue Juliette Wytsmanstraat 14, 1050 Brussels, Belgium; 2 Unit of Epidemiology and Social Medicine, University of Antwerp, Antwerp, Belgium

**Keywords:** Self-administered questionnaire, Web, Mixed-mode, Unit response, Item non-response, Health interview survey

## Abstract

**Background:**

Many population health surveys consist of a mixed-mode design that includes a face-to-face (F2F) interview followed by a paper-and-pencil (P&P) self-administered questionnaire (SAQ) for the sensitive topics. In order to alleviate the burden of a supplementary P&P questioning after the interview, a mixed-mode SAQ design including a web and P&P option was tested for the Belgian health interview survey.

**Methods:**

A pilot study (*n* = 266, age 15+) was organized using a mixed-mode SAQ design following the F2F interview. Respondents were invited to complete a web SAQ either immediately after the interview or at a later time. The P&P option was offered in case respondents refused or had previously declared having no computer access, no internet connection or no recent usage of computers. The unit response rate for the web SAQ and the overall unit response rate for the SAQ independent of the mode were evaluated. A logistic regression analysis was conducted to explore the association of socio-demographic characteristics and interviewer effects with the completed SAQ mode. Furthermore, a logistic regression analysis assessed the differential user-friendliness of the SAQ modes. Finally, a logistic multilevel model was used to evaluate the item non-response in the two SAQ modes while controlling for respondents’ characteristics.

**Results:**

Of the eligible F2F respondents in this study, 76% (107/140) agreed to complete the web SAQ. Yet among those, only 78.5% (84/107) actually did. At the end, the overall (web and P&P) SAQ unit response rate reached 73.5%. In this study older people were less likely to complete the web SAQ. Indications for an interviewer effect were observed as regard the number of web respondents, P&P respondents and respondents who refused to complete the SAQ. The web SAQ scored better in terms of user-friendliness and presented higher item response than the P&P SAQ.

**Conclusions:**

The web SAQ performed better regarding user-friendliness and item response than the P&P SAQ but the overall SAQ unit response rate was low. Therefore, future research is recommended to further assess which type of SAQ design implemented after a F2F interview is the most beneficial to obtain high unit and item response rates.

## Background

A number of national health interview surveys (HISs) such as those carried out in Belgium, Denmark, the United Kingdom and France included, in addition to a face-to-face (F2F) interview, a paper-and-pencil (P&P) self-administered questionnaire (SAQ) for a specific subsection of the questionnaire [1–4]. In this type of mixed-mode design the SAQ is typically used for sensitive topics such as substance use and mental health in order to reduce potential social desirability bias and to enhance the respondents’ privacy [5]. Nevertheless, completing a P&P SAQ immediately after a long and fatiguing F2F session can seem too burdensome for the respondents and therefore cause a drop in the response rate of the SAQ. Indeed, both the Belgian health interview survey (BHIS) and the Danish HIS report a substantial portion of respondents that fail to complete this additional P&P SAQ [2, 6]. Declining response rates are of general concern for public health researchers. Although, low response rates do not by default lead to high non-response error (i.e. non-respondents who differ substantially on the variables of interest compared to respondents) [7], high response rates are still considered to usually minimize the risk for this error [8]. Furthermore, the declining response rates and the growing complexity regarding the reasons for non-responding challenge the ability to confidently obtain population-representative net samples that provide estimates that are generalizable for the whole population [9].

To keep up with technological advancements and alleviate the burden of lengthy questioning, a mixed-mode SAQ design was tested in the framework of the BHIS, including a web SAQ that can be completed at a more convenient moment for the respondent. Allowing F2F respondents to postpone the SAQ completion can also take place with a P&P SAQ, but this entails the inconvenience for the interviewer of having to collect the P&P SAQ at the respondent’s house or for the respondent of having to mail it back. Neither of these charges occur with the web SAQ, since the data is available immediately and processed automatically.

Besides, implementing web instead of P&P SAQs can have some advantages regarding the quality of the collected data. Computerized surveys such as web SAQs can favor more honest responses to questions about sensitive and socially undesirable behaviors than P&P SAQs. This effect is even more pronounced when respondents can complete the SAQ on their own, without fearing that other household members see their responses [10]. In addition, web SAQs can in comparison with P&P SAQs produce higher data quality in terms of absence of data entry mistakes and reduction in the amount of inconsistent and abnormal answers [11, 12]. Furthermore, the level of item non-response generally tends to be lower in web SAQs [11–15]. Indeed, web SAQs present the advantage of automatic data entry, integrated warning messages in case of missing, inconsistent and out-of-range answers and automatic branching logic.

Giving respondents the opportunity to complete the web SAQ at a more convenient moment, i.e. not immediately after the F2F interview, is also less cognitively demanding. This could help decrease satisficing behavior, that is, simply providing satisfactory answers instead of making the effort to generate optimal answers [16]. Nevertheless, it should be acknowledged that P&P SAQs also have some advantages over web SAQs, as the latter offer a greater opportunity to multitask since respondents are more likely to be involved in several other activities while completing the questionnaire [17, 18]. Additionally, web SAQs may limit the ability of the respondents to re-read the questions on their own pace, reply in their preferred order and synchronize their answers [18, 19].

Finally, web SAQs cannot substitute P&P SAQs as the sole mode of data collection because of under-coverage: even in countries with a high internet penetration rate, access still varies among socio-demographic groups in favor of younger, higher educated, higher-income and employed inhabitants [20]. Therefore, both a web and P&P SAQ need to be integrated in a mixed-mode design. In this design the SAQ can be introduced either concurrently, whereby respondents are given a choice of modes, or sequentially, whereby the web SAQ is first introduced alone and the P&P mode is only offered in case the respondent is reluctant to use a web SAQ. There is evidence from stand-alone SAQ surveys that sequential designs introducing the web SAQ first lead to more people responding via web (i.e. a higher web response rate) compared to concurrent designs [15, 21, 22]. Likewise, sequential designs can gain higher overall response rates in comparison with concurrent designs [21]. However, the available evidence regarding unit response rates in mixed-mode designs comes from stand-alone SAQs. These studies do not include a F2F interview and often use different sampling procedures and survey topics. For this reason, their results cannot be generalized to predict the unit response rates in a sequential mixed-mode design for a SAQ as part of a general population HIS.

Hence, this pilot study uses the BHIS survey as a framework to assess the acceptability and the feasibility of a mixed-mode SAQ design within a F2F survey. Different indicators can account for this assessment: the proportion of respondents opting for the web SAQ after completing the F2F interview, the unit response rate for the web SAQ and the overall unit response rate for the SAQ independent of the mode. Secondly, the determinants associated with completing the web SAQ instead of the P&P SAQ are explored. Thirdly, a series of questions at the end of the SAQ address the experience of respondents with the web or the P&P modes, and their respective ease of use is compared. Lastly, in order to assess the quality of the collected data, the relationship between the mode of SAQ completion and the level of item non-response is analyzed. Based on the results of this pilot study, recommendations are formulated for future studies that are needed to further assess the implications of mixed-mode SAQ designs within F2F interviews.

## Methods

### Study design

This pilot study is a cross-sectional epidemiologic survey conducted in the general population and comparable to the BHIS in terms of the sampling, study population, fieldwork and content of the questionnaires.

The BHIS is organized every 4 to 5 years and collects health information from around 10,000 individuals in a F2F setting. The study is organized on household level and maximum 4 individuals are selected per household. The F2F interviews are supplemented with a P&P SAQ covering more sensitive topics. This SAQ is only addressed to the participants of 15 years and older who do not have their F2F interview completed by proxy. Completing the F2F interview takes on average between 30 to 60 min and completing the P&P SAQ takes on average between 20 to 40 min. More details on the survey methodology applied in the BHIS are described elsewhere [1].

#### Sampling and study population

Our pilot study conducted to assess a mixed-mode SAQ design for the BHIS was also organized on household level. The target population in this study consisted of individuals aged 15 years and older residing in Belgium and the National Public Register was used as the sampling frame. Households were selected following a multistage clustered sampling design, involving a geographical stratification by region and municipality of residence. Two municipalities were selected in each Belgian region (Flemish, Brussels-Capital & Walloon Region) based on the degree of urbanization, the socio-economic level and the preferences of the experienced BHIS interviewers (proximity, earlier interviews done in this municipality, etc.). Each interviewer was active in one of the six defined municipalities. Within each municipality, households were selected through a systematic sampling based on the age group of the reference person (< 45, 45–64, > 64) and the household size (1, 2, 2+). The net sample size was 266 individuals. Maximum 4 individuals were selected per household.

#### Data collection

All selected households received an invitation letter that briefly described the purpose, the content and the voluntary character of the pilot study. Furthermore, it stated that an interviewer would visit the household for the F2F interview. The F2F interview was conducted via CAPI (Computer Assisted Personal Interview). Proxy interviewing (one person -either a member of the household or somebody else- was allowed to respond on behalf of the selected person) was conducted for selected household members not able to respond themselves, for those who refused to respond personally but agreed to have a proxy respond for them and for those absent during the time of the F2F interview.

A sequential design was used to introduce the SAQ to the respondents. At the end of the F2F interview, participants were asked questions concerning their computer access, internet connection at home and internet use through a computer in the past 30 days. Those who gave a negative answer to one of these questions were automatically given the P&P SAQ. Those who answered positively to these questions were considered eligible for the web SAQ and were asked to complete it online (either during the interview session or later) while the option of the P&P SAQ was only proposed in case of an explicit refusal. Those who were absent during the interview and had the F2F questionnaire completed via proxy interviewing, received an instruction form to access and answer the web SAQ at a later stage. However, this option was only applicable if the proxy confirmed to the interviewer that the person absent during the F2F session was capable of completing a web SAQ and had access to internet and a computer. Finally, those who agreed to complete the web SAQ but failed to do so, received a reminder letter one week after the F2F interview and a second reminder letter two weeks after the F2F interview.

#### Web SAQ

The web SAQ was developed using Blaise® Internet Service (BlaiseIS) software and could be completed using a computer but not using a tablet or smartphone. The appearance of the web version was very similar to the one of the P&P SAQ. The questions were identical (similar wording and almost similar instructions) and the design was comparable (similar colors and lay-out). Still, it was developed while applying the imbedded features of this mode such as automatic skipping and branching. Furthermore, soft warnings were given in case of missing values for the first question of every module of the questionnaire, for filter questions and in case respondents gave inconsistent or implausible answers. Finally, the web SAQ had a multipage design displaying only a few questions on every screen which differs from the P&P SAQ that allows a more comprehensive view on the whole questionnaire. Web respondents were able to go back in the questionnaire to change answers given to previous questions.

### Statistical analyses

First, descriptive summary statistics were provided in order to outline the characteristics of the F2F respondents by completed SAQ mode. In addition, a multinomial logistic regression analysis was conducted to assess, among F2F respondents, the association of the socio-demographic characteristics of the respondents (i.e. age, sex, education level, employment status and household size) and the interviewer involved with the SAQ mode completed.

Second, a description of the proportion of respondents who found the SAQ easy to use and who experienced no problems was given by completed SAQ mode. In addition, a logistic regression analysis was conducted to assess the association between the SAQ mode used & the socio-demographic characteristics of the respondents (i.e. age, sex, education level, employment status and household size) and their evaluation of the SAQ’s user-friendliness.

Third, it was assessed whether item non-response is related to the SAQ mode or to the characteristics of the respondents. Since the data can be viewed as hierarchical with items as the lowest level and respondents as the highest level, a logistic multilevel model was used (dichotomous dependent variable: 1 = no answer, 2 = answer). More specifically, we used a 2-level Generalised Linear Mixed Model with random intercepts only. Respondents with over 50% of items missing were classified as partial respondents and were excluded from the item non-response analysis. The model building process was performed in three steps. First, an intercept-only multilevel model was fitted (model 1). In the second step, mode was added as a fixed effect to assess the effect of the level 1 variable (model 2). In the final step, the model building process was continued by adding respondent characteristics to model 2 in order to separate mode effects from selection effects (model 3).

The intra-class correlation coefficient (ICC) was calculated to provide information on how much of the variation in item non-response was accounted for by respondent characteristics [23]. We checked for significant variances with the Wald test [24] and calculated the proportional change in variance (PCV) between the initial model and the models with more terms [25]. The SAS® Glimmix procedure with Laplace estimation method was used to identify if mode and respondent characteristics are associated with item non-response and to estimate the variance associated with each level. Associations were expressed as odds ratios (OR) with 95% confidence interval (CI). As all the models are nested and as maximum likelihood estimation based on Laplace approximation was used, likelihood ratio (deviance) tests were conducted to compare the relative fit of the different models [23]. The difference in deviance of two nested models has a chi-square distribution with degrees of freedom equal to the difference in the number of parameters between the models. All statistical analyses were carried out using SAS® Enterprise Guide 5.1.

## Results

### Study flow

**Fig. 1:**
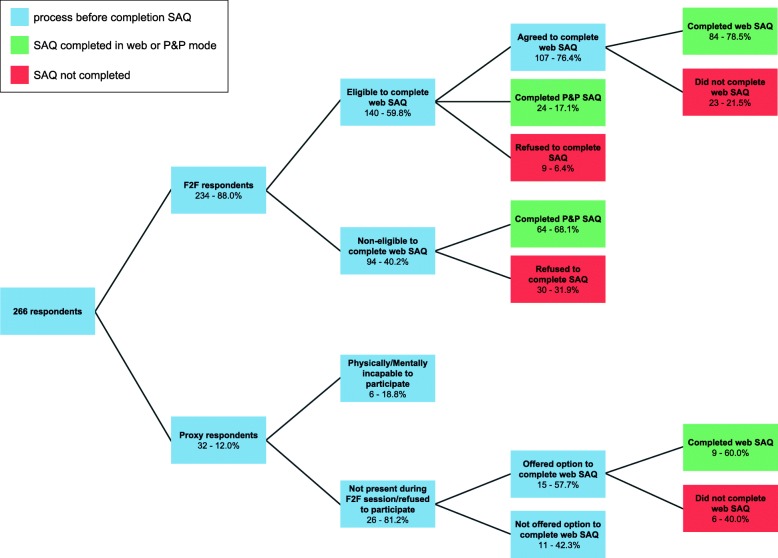
Study diagram. F2F = face-to-face; P&P = paper-and-pencil; SAQ = self-administered questionnaire

A detailed overview of the study flow is given in Fig. 1. Among the F2F respondents, 59.8% were considered as eligible for the SAQ by web. Of these, 76.4% agreed to complete the web SAQ, 17.1% refused but agreed to complete the P&P SAQ instead and 6.4% refused to complete the SAQ in both modes. Nevertheless, among the F2F respondents who agreed to complete the web SAQ, 21.5% did not complete it even after two reminder letters were sent. In total, 172 of the 234 (73.5%) F2F respondents completed the SAQ in either the web (84/172–48.8%) or P&P (88/172–51.2%) mode. Among the respondents with a proxy F2F interview who received the option to complete the web SAQ, eventually 60.0% completed it.

### Completion by SAQ mode

Table 1 shows the characteristics of the respondents according to the mode of SAQ completion. Web respondents were more likely to be young, higher educated and employed. Additionally, large differences in the proportion of respondents in the web, P&P and refusal group could be found between interviewers. For instance, two interviewers had more than half of their respondents completing the web SAQ whereas one interviewer had only one person (2.4%) opting for web. Furthermore, the proportion of respondents who refused to complete the SAQ varied substantially among interviewers (from 5 to 55%).

**Table 1:** Descriptive overview of F2F respondents according to the mode of SAQ completion (*n* = 234)

	Web (*n* = 84)		P&P (*n* = 88)		Refusal (*n* = 62)	
Respondent characteristics						
	Mean	SD	Mean	SD	Mean	SD
Age	46.0	15.6	56.5	20.9	48.9	18.2
	N	%	N	%	N	%
Sex						
men (*n* = 103)	36	34.9	38	36.9	29	28.2
women (*n* = 131)	48	36.6	50	38.2	33	25.2
Education level ^a^						
lower (*n* = 154)	46	29.9	63	40.9	45	29.2
higher (*n* = 80)	38	47.5	25	31.3	17	21.3
Country of birth						
Belgium (*n* = 174)	70	40.2	68	39.1	36	20.7
other countries (*n* = 60)	14	23.3	20	33.3	26	43.3
Employment status ^b^						
employed (*n* = 101)	50	49.5	25	24.8	26	25.7
unemployed (*n* = 110)	25	22.7	54	49.1	31	28.2
student (n = 23)	9	39.1	9	39.1	5	21.7
Household size						
1 (n = 84)	26	31.0	38	45.2	20	23.8
1+ (*n* = 150)	58	38.7	50	33.3	42	28.0
Interviewer						
	N	%	N	%	N	%
Interviewer 1 (*n* = 29)	8	27.6	16	55.2	5	17.2
Interviewer 2 (*n* = 49)	19	38.8	20	40.8	10	20.4
Interviewer 3 (*n* = 47)	19	40.4	13	27.7	15	31.9
Interviewer 4 (*n* = 37)	21	56.8	14	37.8	2	5.4
Interviewer 5 (*n* = 30)	16	53.3	7	23.3	7	23.3
Interviewer 6 (*n* = 42)	1	2.4	18	42.9	23	54.8

F2F = face-to-face, SAQ = self-administered, P&P = paper-and-pencil.

^a^ For students the education level equals the highest education level in the household. If this was not possible it was based on their current field of study

^b^ “Employed” refers to respondents who had a paid job for at least one hour a week at the moment of the interview, “Unemployed” refers to respondents without a paid job at the moment of the interview, “Student” refers to respondents who were (fulltime or part-time) enrolled in the regular education system at the moment of the interview. Students who had a (part-time) paid job were classified as students

Table 2 shows that only age of the respondent was significantly associated with the mode used to complete the SAQ in this pilot study; older people were more likely to complete the P&P SAQ instead of the web SAQ (OR (95% CI): 1.04 (1.01–1.07)). In addition, the completed SAQ mode of the F2F respondents is associated with the interviewers. Interviewers 3 (OR (95% CI): 0.28 (0.08–0.96)) and 5 (OR (95% CI): 0.14 (0.03–0.62)) had significantly less respondents who completed the SAQ using P&P versus using the web in comparison with the reference interviewer. Interviewer 4 had fewer respondents who did not complete the SAQ in any mode versus web respondents (OR (95% CI): 0.15 (0.02–0.93)) in comparison with the reference interviewer.

**Table 2:** Association of socio-demographic characteristics and interviewer with the mode of SAQ completion (*n* = 192)

	P&P	Refusal
	OR (95% CI)	OR (95% CI)
Age	1.04 (1.01–1.07) *	1.00 (0.97–1.03)
Men	1.36 (0.65–2.84)	0.97 (0.43–2.17)
Higher education level ^a^	0.89 (0.37–2.13)	1.20 (0.47–3.04)
Country of birth: non-Belgium	1.15 (0.37–3.55)	0.89 (0.25–3.13)
Employment status (ref: non-employed) ^b^		
employed	0.56 (0.21–1.51)	0.87 (0.29–2.64)
student	2.13 (0.35–12.99)	1.34 (0.20–8.80)
Household size: 1+	0.51 (0.24–1.10)	0.66 (0.29–1.54)
Interviewer (ref: interviewer 1) ^c^		
Interviewer 2	0.52 (0.16–1.64)	0.84 (0.21–3.32)
Interviewer 3	0.28 (0.08–0.96) *	1.30 (0.34–4.99)
Interviewer 4	0.32 (0.10–1.05)	0.15 (0.02–0.93)*
Interviewer 5	0.14 (0.03–0.62) *	0.60 (0.12–2.98)
Nagelkerke R^2^	0.28 *	

A multinomial logistic regression model using SAS Proc Logistic with web SAQ as reference category

^*^p value < 0.05; SAQ = self-administered questionnaire; P&P = paper-and-pencil

^a^ For students the education level equals the highest education level in the household. If this was not possible it was based on their current field of study

^b^ “Employed” refers to respondents who had a paid job for at least one hour a week at the moment of the interview, “Unemployed” refers to respondents without a paid job at the moment of the interview, “Student” refers to respondents who were (fulltime or part-time) enrolled in the regular education system at the moment of the interview. Students who had a (part-time) paid job were classified as students

^c^ Interviewer 6 had a refusal rate of 54.8% for the SAQ among the F2F respondents and had only 1 of 42 (2.4%) F2F respondents who completed the web SAQ. Therefore, this interviewer was considered as an outlier and was not considered in this analysis

### User-friendliness of the web versus P&P SAQ

Of the web respondents, 87.9% (80/91) thought the SAQ was easy to use while this was only the case for 63.5% (54/85) of the P&P respondents. In line with these results, more P&P respondents (14.3% - 12/84) declared experiencing problems while completing the SAQ than web respondents (7.8% - 7/90). Even after controlling for socio-demographic characteristics, more web respondents than P&P respondents evaluated the SAQ as being easy to use (OR (95% CI): 2.55 (1.08–6.04)).

### Item non-response

Among the SAQ respondents, 16 (8.8%) were considered as partial respondents as they completed less than 50% of the items in the SAQ and they were therefore dropped from the item non-response analysis. The number of partial respondents was significantly higher in the P&P SAQ than in the web SAQ (P&*P* = 15.9% vs. web = 2.1%; *p* = 0.0011). Additionally, there were significant differences in the number of partial P&P respondents per interviewer. For example, one interviewer had almost 70% partial respondents whereas others had none (*p* < 0.0001). In the respondents that completed more than 50% of the SAQ items, there was an item non-response of 6.0% in total for the SAQ. Item non-response was still significantly higher in the P&P SAQ than in the web SAQ (P&*P* = 9.2% vs. web = 3.3%; *p* < 0.0001).

The results of the multilevel logistic regression analysis for item non-response are presented in Table 3. The first column presents the results of the empty model and shows that approximately 37% of the variability in item non-response is accounted for by respondents, leaving 63% of the variability accounted for by the mode or other unknown factors. This means that, in general, mode or other unknown factors are more important than respondent characteristics. Adding mode to the model (model 2) decreased the respondent variance and explained 11.4% of this variance in the empty model. Together the mode and respondent characteristics explained almost 13.6% of all respondent variance (model 3).

**Table 3:** Multilevel model for item non-response in SAQ (*n* = 21,763)

	Model 1 (Empty model)	Model 2	Model 3^a^
	OR (95% CI)	OR (95% CI)	OR (95% CI)
Fixed effects at item level			
Mode: P&P		2.25 (1.44–3.52) *	2.13 (1.33–3.43) *
Fixed effects at respondent level			
Age			1.00 (0.98–1.02)
Men			0.81 (0.52–1.28)
Higher education level ^b^			0.79 (0.47–1.33)
Country of birth: non-Belgium			1.00 (0.55–1.81)
Employment status (ref: non-employed) ^c^			
employed			0.91 (0.48–1.71)
student			0.84 (0.31–2.26)
Household size: 1+			0.97 (0.59–1.58)
Random parameters			
Variance between respondents (SE)	1.92 (0.28) *	1.70 (0.25) *	1.66 (0.25) *
ICC _respondents_	0.37	0.34	0.34
PCV		11.4%	13.6%
Model fit: −2 Log Likelihood	7840.89	7829.08 **	7826.66

Values based on SAS Proc Glimmix; Estimation method = Laplace

^*^p value < 0.05; ** = likelihood ratio test significant; ICC = intra-class coefficient of correlation; PCV = proportional change in variance; SAQ = self-administered; P&P = paper-and-pencil

The PCV expresses the change in the respondents’ level variance between the initial model and the model with more terms.

^a^ Best fitting model

^b^ For students the education level equals the highest education level in the household. If this was not possible it was based on their current field of study

^c^ “Employed” refers to respondents who had a paid job for at least one hour a week at the moment of the interview, “Unemployed” refers to respondents without a paid job at the moment of the interview, “Student” refers to respondents who were (fulltime or part-time) enrolled in the regular education system at the moment of the interview. Students who had a (part-time) paid job were classified as students

The results of Table 3 indicate that the mode in which the SAQ was completed had a significant impact on the item non-response, also after controlling for differences in item non-response among respondents; respondents completing the P&P SAQ had a higher level of item non-response than respondents completing the web mode (OR (95% CI): 2.13 (1.33–3.43)).

## Discussion

Taking advantage of the technological advancements applied to data collection, a web SAQ to be completed after the F2F interview was pilot tested in the framework of the BHIS under the assumption that it reduces the burden for the F2F respondents and improves the data quality with regard to the traditional P&P SAQ. However, since web SAQs cannot be used as the sole mode of data collection in representative population surveys, it was integrated in a mixed-mode design (web or P&P). The importance of using a mixed-mode design is shown by the fact that only 60% of the F2F respondents were eligible to complete the web SAQ. This is an indication that using web-only surveys still excludes parts of the population who do not have internet access or who are not computer-literate [26]. Furthermore, it confirms that mixed-mode data collection should still be preferred over web-only data collection for population-based health surveys organized in Belgium [27].

The first goal of this study was to test the feasibility of organizing a sequential mixed-mode SAQ in terms of unit response. Among the eligible F2F respondents (regular PC users with internet connection), a substantial proportion (76%) agreed to complete the web SAQ. This was expected as the interviewer first requested responding via web SAQ, while withholding the P&P option until later in the implementation phase. Evidence from stand-only SAQ surveys shows that initially inviting people (either by e-mail or by postal mail) to complete a web SAQ and only offering a P&P SAQ to the non-respondents, is highly successful in making people participate online instead of on P&P [15, 28].

Nevertheless, among the F2F respondents in the pilot study who agreed to complete the web SAQ, almost 22% did not complete it, despite sending two reminder letters. This finding suggests that inviting respondents to complete a web SAQ in a second stage after the F2F interview (i.e. not during the interview session) might have a negative impact on the response. Second stage non-response is also of concern in other health surveys. At the end of the F2F or telephone interview, respondents of the Swiss health survey were invited to complete an additional P&P SAQ which was sent to them 2–3 days after the interview [29]. Among these respondents 80% returned the SAQ. Likewise, Akmatov et al. [30] obtained a second-stage response of 61% for a web survey organized after a health examination survey in Germany.

Finally, 26% of the F2F respondents did not complete the SAQ in any mode compared to only 18% in the BHIS edition of 2013 [6]. This result could imply that a sequential mixed-mode design does not increase the SAQ response in comparison with a P&P-only design for F2F respondents. The lower SAQ response rate found in this pilot study could partially be explained by the fact that a substantial amount of F2F respondents who agreed to complete the web SAQ after the interviewer left their house did not do so. Furthermore, also mixed-mode designs applied in stand-alone surveys whereby respondents are first mailed to complete a web SAQ and only in case of non-response a P&P SAQ is mailed can produce lower response rates than P&P-only surveys [31, 32]. Nevertheless, the lower SAQ response rate found in this study compared to the SAQ response rate of the BHIS 2013 could also partly find an explanation in the general decrease in response rates for epidemiologic studies [9, 33].

In our study, among the individuals who were not present at the moment of the interview (i.e. proxy interviews) and who received the option to complete the web SAQ, 60% actually complied. It should be acknowledged that these respondents did not receive the same stimuli to participate than the F2F respondents, as instead of the interviewer, another household member gave the instruction form to complete the web SAQ and as their response burden was much lower as they did not complete the F2F interview. Nevertheless, this finding illustrates that a web SAQ option might possibly be a strategy to decrease the SAQ non-response due to the proxy respondents.

When considering all the F2F respondents (that is the ones eligible and non-eligible for web SAQ completion), we found that older people were less likely to complete a web SAQ instead of a P&P SAQ. However, none of the other socio-demographic characteristics were significantly associated with the completed SAQ mode in this pilot study. Previous mixed-mode studies also showed that elderly are less likely to choose the web mode over the P&P mode than their younger counterparts [28, 34]. This can be explained by the fact that elderly are less likely to have internet access [35–37] and use the internet on a less frequent basis [38]. Indeed, an additional analysis indicated that F2F respondents older than 65 were substantially less likely to be eligible to complete the SAQ by web than the younger F2F respondents. Nevertheless, we can expect that especially in these older age groups the preference for the web mode will (further) increase in time [28].

The results show potential indications for an interviewer effect as the involved interviewers differed in their share of web SAQ respondents, P&P SAQ respondents and respondents who refused to complete the SAQ. Some interviewers had also more P&P respondents who only filled out the SAQ partially. These findings could suggest that interviewers play a crucial role in gaining cooperation for the SAQ, in the type of SAQ mode completed and in the data quality of the completed SAQ. The first finding is in line with the results of a previous BHIS study that found a substantial interviewer’s impact on the non-response for the P&P SAQs as the interviewer has to maintain the participants’ motivation to continue with the SAQ [6]. Interviewers contribute to survey error as they do not always strictly follow the standardized interviewer techniques resulting in incorrect skipping of questions, incorrect reading of questions (i.e. shortening or rewording), incorrect probing, etc. [39]. This study adds that interviewers might also vary in the way in which they follow the proposed study guidelines (e.g. convincing respondents to complete the SAQ, persuading respondents to complete the web SAQ and motivating P&P respondents to complete the SAQ properly).

Web respondents in this study found more often than P&P respondents that the SAQ was user-friendly, which is similar to findings from other studies [11, 40]. This could be related to the fact that more guidance is provided in the web SAQ than in the P&P SAQ (for example due to the automatic branching logic). Furthermore, in this study it could also be due to the fact that web respondents were less likely to complete the SAQ during the already long and fatiguing F2F session.

Our results showed that the web SAQ performed better regarding completeness of the data in comparison with the P&P SAQ. First, there were less partial respondents among the web respondents which may be due to the fact that they could postpone the completion of the SAQ at their best convenience, while the P&P SAQ had to be completed just after the lengthy F2F interview. As a consequence, the web mode might have had a lower response burden in comparison with the P&P mode. A lower perceived response burden and continued interest in the questionnaire have also been previously identified as factors to fill out web questionnaires until the end [41]. Second, the web SAQ performed better in terms of item response. This could also be related to the lower response burden experienced by the web respondents and to the multiple design features that were integrated in the web mode (automatic branching logic, individual page construction and warning messages in case of missing answers for some questions) which were not part of the P&P mode. This finding is in line with previous studies that generally found that electronic data collection modes have lower item non-response rates in comparison with traditional P&P modes [11–15].

Limitations of this pilot study need to be acknowledged, most notably the lack of power of this study as a result of the small sample size (*n* = 266). For example, although there were indications that higher educated and employed F2F respondents were more likely to complete the web SAQ, our small sample size did not allow to draw any conclusions on this. The design of this study was also specific: a sequential mixed-mode design (web or P&P) as a part of a F2F survey. For these reasons caution should be exercised when extrapolating the results to the general population and to other types of mixed-mode designs for SAQs. Furthermore, during the feedback session with the interviewers we noticed that one interviewer who is experienced with conducting interviews for the BHIS was very reluctant to switch to a mixed-mode design including web for the SAQ. For this reason, a sensitivity analysis without the respondents of this interviewer was conducted to check whether the same conclusions regarding the agreement to complete the web SAQ and the SAQ unit response rate would hold. The results indicated that among the respondents eligible to do the web SAQ more (91% instead of 76%) would have opted for web. Furthermore, the overall response rate would be slightly higher (80% instead of 74%) since this interviewer had a high refusal rate for the SAQ.

Besides, in this study results point to the strong impact of the interviewers, nevertheless, this study did not allow disentangling the interviewer effect from the neighborhood effect as interviewers were mainly active in one municipality. One can assume that people living in the same neighborhood could be more or less incline to complete a web SAQ and to do better or worse regarding item response. Nevertheless, the study of Berete et al. [6] based on the BHIS 2013 tested whether interviewer variability in non-response for the P&P SAQ reflected neighborhood variability using a multi-level model and concluded that this was not the case. Finally, due to the complex sampling design of the BHIS which contains stratification and clustering at different level points and variance, estimates are biased if design effects are not taken into consideration during data analyses [1]. Nevertheless, for this study, no correction methods were applied in order to adjust for intra-municipality or intra-household correlation.

A strength of this study is the usage of a multilevel model to assess item non-response since the data can be viewed as hierarchical with items at the lowest and respondents at the highest level. Analyzing data at any of these levels while ignoring the more detailed level or higher level can lead to incorrect conclusions [23]. A similar two-level model was used by Borgers and Hox [42] in order to test the association of item characteristics and respondent characteristics on item non-response in a questionnaire for children.

## Conclusion

The purpose of this pilot study was to explore the feasibility of a mixed-mode design for the SAQ (web or P&P) of the BHIS in terms of unit and item (non-) response and user-experience. A substantial number of respondents had accepted to complete the web SAQ but this type of mixed-mode SAQ design did not increase the SAQ response rate in comparison with the P&P-only SAQ applied in the BHIS 2013. This could partially be explained by the substantial number of respondents who agreed to complete the web SAQ but did not complete it despite the strict follow-up. Nevertheless, the web SAQ was more user-friendly and scored better in terms of item response than the P&P SAQ. Finally, this study found some indications that interviewers play an important role in gaining cooperation for the SAQ, in the choice of SAQ mode and in the data quality of the completed SAQ.

As a consequence of the low unit response rate found in this pilot study, a mixed-mode SAQ design including a web mode to be completed later was not implemented in the latest BHIS edition (2018). However, as this web SAQ did show advantages in terms of perceived user-friendliness and item response, we are further experimenting with web-based data collection in the context of the BHIS.

In accordance with this, we cannot currently advise researchers involved in other F2F surveys containing a SAQ for a part of the questionnaire, to switch from a P&P SAQ to a mixed-mode SAQ including a web mode. More research is needed. We encourage organizing research to assess which type of SAQ designs implemented after conducting a F2F interview is the most beneficial to obtain both high unit and item response rates for the SAQ. Such research should examine which socio-demographic and survey contextual factors influence the completion of the (web) SAQ. In order to do so, a possible design would be an experiment whereby respondents are randomly assigned to one of the following SAQ designs after completing the F2F survey: 1) a P&P-only SAQ, 2) a concurrent P&P - web SAQ, 3) a sequential web > P&P SAQ and 4) a CASI (Computer Assisted Self-Interviewing) questionnaire integrated within the CAPI itself. These experimental survey designs should involve larger sample sizes and more interviewers.

## Data Availability

The datasets used and/or analysed during the current study are available from the corresponding author on reasonable request.

## Abbreviations

BHIS: Belgian Health Interview Survey
CAPI: Computer Assisted Personal Interview
CASI: Computer Assisted Self-Interviewing
CI: Confidence Interval
F2F: Face-To-Face
HIS: Health Interview Survey
ICC: Intra-class Correlation Coefficient
OR: Odds Ratio
P&P: Paper-and-Pencil
PCV: Proportional Change in Variance
SAQ: Self-Administered Questionnaire
SD: Standard Deviation

## References

[1] Demarest S, Van der Heyden J, Charafeddine R, Drieskens S, Gisle L, Tafforeau J (2013). Methodological basics and evolution of the Belgian health interview survey 1997-2008. Arch Public Health.

[2] Ekholm O, Hesse U, Davidsen M, Kjoller M (2009). The study design and characteristics of the Danish national health interview surveys. Scand J Public Health.

[3] Mindell JS, Tipping S, Pickering K, Hope S, Roth MA, Erens B (2010). The effect of survey method on survey participation: analysis of data from the health survey for England 2006 and the boost survey for London. BMC Med Res Methodol.

[4] Célant N, Rochereau T. l’Enquête santé européenne-Enquête santé et protection sociale (EHIS-ESPS) 2014 [The European health interview survey - Health and social protection survey (EHIS-ESPS) 2014]. In: Rapport IRDES °566. 2017. http://www.irdes.fr/recherche/rapports/566-enquete-sante-europeenne-ehis-enquete-sante-et-protection-sociale-esps-2014.pdf. Accessed 18 Oct 2019.

[5] De Leeuw ED, Hox JJ, Das M, Ester P, Kaczmirek L (2011). Internet surveys as part of a mixed-mode design. Social and behavioral research and the Internet: Advances in applied methods and new research strategies.

[6] Berete F, Van der Heyden J, Demarest S, Charafeddine R, Gisle L, Braekman E (2019). Determinants of unit nonresponse in multi-mode data collection: a multilevel analysis. PLoS One.

[7] Groves RM, Peytcheva E (2008). The impact of nonresponse rates on nonresponse bias: a meta-analysis. Public Opin Q..

[8] Daikeler J, Bošnjak M, Manfreda KL (2019). Web versus other survey modes: an updated and extended meta-analysis comparing response rates. J Surv Stat Methodol.

[9] Galea S, Tracy M (2007). Participation rates in epidemiologic studies. Ann Epidemiol.

[10] Gnambs T, Kaspar K (2015). Disclosure of sensitive behaviors across self-administered survey modes: a meta-analysis. Behav Res Methods.

[11] Touvier M, Méjean C, Kesse-Guyot E, Pollet C, Malon A, Castetbon K (2010). Comparison between web-based and paper versions of a self-administered anthropometric questionnaire. Eur J Epidemiol.

[12] Vergnaud AC, Touvier M, Méjean C, Kesse-Guyot E, Pollet C, Malon A (2011). Agreement between web-based and paper versions of a socio-demographic questionnaire in the NutriNet-Santé study. Int J Public Health..

[13] Bowling A (2005). Mode of questionnaire administration can have serious effects on data quality. J Public Health.

[14] Kwak N, Radler B (2002). A comparison between mail and web surveys: response pattern, respondent profile, and data quality. J Off Stat.

[15] Mauz E, von der Lippe E, Allen J, Schilling R, Müters S, Hoebel J, et al. Mixing modes in a population-based interview survey: comparison of a sequential and a concurrent mixed-mode design for public health research. Arch Public Health. 2018;76:8. 10.1186/s13690-017-0237-1 PMC5791202 29423220

[16] Krosnick JA (1991). Response strategies for coping with the cognitive demands of attitude measures in surveys. Appl Cogn Psychol.

[17] Fang J, Prybutok V, Wen C (2016). Shirking behavior and socially desirable responding in online surveys: a cross-cultural study comparing Chinese and American samples. Comput Hum Behav.

[18] Gwaltney CJ, Shields AL, Shiffman S (2008). Equivalence of electronic and paper-and-pencil administration of patient-reported outcome measures: a meta-analytic review. Value Health.

[19] Shim JM, Shin E, Johnson TP (2013). Self-rated health assessed by web versus mail modes in a mixed mode survey: the digital divide effect and the genuine survey mode effect. Med Care.

[20] Bethlehem J, Engel U, Jann B, Lynn P, Scherpenzeel A, Sturgis P (2015). Web surveys in official statistics. Improving survey methods: lessons from recent research.

[21] Millar MM, Dillman DA (2011). Improving response to web and mixed-mode surveys. Public Opin Q.

[22] Werner P, Forsman G. Mixed mode data collection using paper and web questionnaires. In: Proceedings of the American Statistical Association, Section on Survey Research Methods. 2005. http://citeseerx.ist.psu.edu/viewdoc/download?doi=10.1.1.576.4749&rep=rep1&type=pdf. Accessed 18 Oct 2019.

[23] Ene M, Leighton EA, Blue GL, Bell BA. Multilevel models for categorical data using SAS PROC GLIMMIX: the basics. In: SAS Global Forum 2015 Proceedings. 2015. http://support.sas.com/resources/papers/proceedings15/3430-2015.pdf. Accessed 18 Oct 2019.

[24] Rosell-Murphy M, Rodriguez-Blanco T, Morán J, Pons-Vigués M, Elorza-Ricart JM, Rodríguez J (2015). Variability in screening prevention activities in primary care in Spain: a multilevel analysis. BMC Public Health.

[25] Merlo J, Chaix B, Ohlsson H, Beckman A, Johnell K, Hjerpe P (2006). A brief conceptual tutorial of multilevel analysis in social epidemiology: using measures of clustering in multilevel logistic regression to investigate contextual phenomena. J Epidemiol Community Health.

[26] McCluskey S, Topping AE (2011). Increasing response rates to lifestyle surveys: a pragmatic evidence review. Perspect Public Health.

[27] Braekman E, Charafeddine R, Demarest S, Drieskens S, Tafforeau J, Van der Heyden J, et al. Is the European Health Interview Survey online yet? Response and net sample composition of a web-based data collection. Eur J Public Health. 2019;ckz206. 10.1093/eurpub/ckz206. 10.1093/eurpub/ckz206 31697353

[28] Jensen HAR, Ekholm O, Davidsen M, Christensen AI (2019). The Danish health and morbidity surveys: study design and participant characteristics. BMC Med Res Methodol.

[29] Volken T (2013). Second-stage non-response in the Swiss health survey: determinants and bias in outcomes. BMC Public Health.

[30] Akmatov MK, Rübsamen N, Schultze A, Kemmling Y, Obi N, Günther K (2015). Diverse recruitment strategies result in different participation percentages in a web-based study, but in similar compliance. Int J Public Health.

[31] Messer BL, Edwards ML, Dillman DA (2012). Determinants of item nonresponse to web and mail respondents in three address-based mixed-mode surveys of the general public. Surv Pract.

[32] Dillman DA (2017). The promise and challenge of pushing respondents to the web in mixed-mode surveys. Surv Methodol.

[33] Morton SMB, Bandara DK, Robinson EM, Carr PEA (2012). In the 21st century, what is an acceptable response rate?. Aust N Z J Public Health.

[34] Zuidgeest M, Hendriks M, Koopman L, Spreeuwenberg P, Rademakers J (2011). A comparison of a postal survey and mixed-mode survey using a questionnaire on patients’ experiences with breast care. J Med Internet Res.

[35] Mohorko A, De Leeuw ED, Hox J (2013). Internet coverage and coverage bias in Europe: developments across countries and over time. J Off Stat.

[36] Leenheer J, Scherpenzeel AC (2013). Does it pay off to include non-internet households in an internet panel?. Int J Internet Sci.

[37] Sterrett D, Malato D, Benz J, Tompson T, English N (2017). Assessing changes in coverage bias of web surveys in the United States. Public Opin Q..

[38] Statbel, ICT-statistieken bij individuen [ICT statistics among individuals], Belgium: https://statbel.fgov.be/nl/themas/huishoudens/ict-gebruik-huishoudens#figures. Accessed 15 Sept 2019.

[39] Ackermann-Piek D, Massing N (2014). Interviewer behavior and interviewer characteristics in PIAAC Germany. Methods Data Analyses.

[40] Naus MJ, Philipp LM, Samsi M (2009). From paper to pixels: a comparison of paper and computer formats in psychological assessment. Comput Hum Behav.

[41] Galesic M (2006). Dropouts on the web: effects of interest and burden experienced during an online survey. J Off Stat.

[42] Borgers N, Hox J (2001). Item nonresponse in questionnaire research with children. J Off Stat.

